# Common Sequences of Emergency Readmissions among High-Impact Users following AAA Repair

**DOI:** 10.1155/2018/5468010

**Published:** 2018-07-03

**Authors:** Ahsan Rao, Alex Bottle, Colin Bicknell, Ara Darzi, Paul Aylin

**Affiliations:** ^1^Dr Foster Unit, Department of Public Health, Imperial College London, 3 Dorset Rise, London EC4Y 8EN, UK; ^2^Department of Surgery, Imperial College London, St. Mary's Hospital, Praed Street, London W2 1NY, UK

## Abstract

**Introduction:**

The aim of the study was to examine common sequences of causes of readmissions among those patients with multiple hospital admissions, high-impact users, after abdominal aortic aneurysm (AAA) repair and to focus on strategies to reduce long-term readmission rate.

**Methods:**

The patient cohort (2006–2009) included patients from Hospital Episodes Statistics, the national administrative data of all NHS English hospitals, and followed up for 5 years. Group-based trajectory modelling and sequence analysis were performed on the data.

**Results:**

From a total of 16,973 elective AAA repair patients, 18% (*n*=3055) were high-impact users. The high-impact users among ruptured abdominal aortic aneurysm (rAAA) repair constituted 17.3% of the patient population (*n*=4144). There were 2 subtypes of high-impact users, short-term (7.2%) with initial high readmission rate following by rapid decline and chronic high-impact (10.1%) with persistently high readmission rate. Common causes of readmissions following elective AAA repair were respiratory tract infection (7.3%), aortic graft complications (6.0%), unspecified chest pain (5.8%), and gastrointestinal haemorrhage (4.8%). However, high-impact users included significantly increased number of patients with multiple readmissions and distinct sequences of readmissions mainly consisting of COPD (4.7%), respiratory tract infection (4.7%), and ischaemic heart disease (3.3%).

**Conclusion:**

A significant number of patients were high-impact users after AAA repair. They had a common and distinct sequence of causes of readmissions following AAA repair, mainly consisting of cardiopulmonary conditions and aortic graft complications. The common causes of long-term mortality were not related to AAA repair. The quality of care can be improved by identifying these patients early and focusing on prevention of cardiopulmonary diseases in the community.

## 1. Introduction

Repair of abdominal aortic aneurysm (AAA) has been associated with very high readmission rates [1]. It is one of the top 7 conditions that account for 30% of all potentially preventable readmissions [1]. With the introduction of screening for AAA at a national level, the number of elective repairs has increased, and the number of repairs for ruptured abdominal aortic aneurysm (rAAA) is expected to decline. However, there are still a significant proportion of patients who suffer from rAAA and undergo repair [2].

The general patient population has a small subgroup of patients, the high-impact users, who have significantly higher rates of unplanned hospitalisations [3]. They are shown to utilise as much as two-thirds of the health-care resources [4]. Risk profiling of the patient population to identify these patients provides health policymakers an opportunity to plan an optimal and individualised patient care by allocating appropriate resources and analysing trends in the health status of a population to prevent decline in the health status at a population level [5]. An increased readmission rate has been associated with higher mortality and discharge to care facility [6]. It may be that there are disease-specific patterns of readmissions in the high-impact users among those with AAA repair. It is important to assess causes of avoidable readmissions and whether these differ from those of the low-impact users.

Understanding the temporal order of the causes of readmissions may help us assess any repeated chain of events that occur in high-impact users. Previous studies have shown that order of events have significant impact on the outcomes of patients [7]. For example, the incidence of heart failure following atrial fibrillation was shown to be associated with a high mortality compared with those patients who were diagnosed with atrial fibrillation after heart failure [8]. What is not certain is whether distinct and common sequences of causes of readmissions are associated with the increase in readmission rate in the high-impact users. The study aimed to assess common causes of readmission and common sequences of causes of readmissions among high-impact users following AAA repair.

## 2. Methods

### 2.1. Hospital Administrative Data

Hospital Episode Statistics (HES) data were used to extract information on patients diagnosed with AAA repair [9]. The data are collected by the Department of Health, Government of England, and include information on all the inpatient hospital admissions of all the patients admitted to public hospitals in NHS (National Health Service), England [9]. All patients, including private ones, who require emergency treatment are initially admitted in these hospitals. Each hospital admission is recorded as a “spell” consisting of “episodes” which denotes the care under each consultant during the patient's stay [10]. If a hospital admission requires a transfer to another hospital before the patient is discharged, then the whole hospital stay is recorded under “superspell” [10]. For the analysis, the information on each patient's spell or superspell was retrieved. All the conditions are coded using ICD-10 classification (International Classification of Diseases version 10). The procedures are coded using OPCS 4.7 (Office of Population Censuses and Surveys Classification of Interventions and Procedures version 4.7) [10].

### 2.2. Patients with AAA Repair

All adult patients over the age of 18 who had primary AAA repair from the year 2006 to 2009 were included in the study. Patients who died during the follow-up period were included in the study as well. The patient cohort comprised two main types of repair, EVAR (endovascular aneurysm repair) and open repair. Initially, specific ICD-10 codes were used to identify AAA patients, as used in previous studies: elective AAA (I714, I719) and ruptured AAA (I713, I718) [11, 12]. Afterwards, the type of repair of AAA was recognised using OPCS 4 codes, as used in earlier studies, and combined with ICD-10 codes to select the patient cohort [13, 14]. The following OPCS codes were used: open repair (L18x, L19x, L20x, L21x, L25x) and EVAR (L26x, L27x, L28x). All the patients were followed up for a minimum of 5 years.

### 2.3. Statistical Methods

The trajectory model was applied to the modified dataset which categorised individuals into different subgroups. The outcome was the annual number of emergency readmissions for each patient for each successive year during the 5-year follow-up. In order to determine the optimum number of subgroups within a population, the choice of model was based on the following criteria: smallest value of Bayesian Information Criteria (BIC), largest value for average posterior probability for each group, odds of correct classification (OCC) >5, and each trajectory with significant parameter estimates (*p* < 0.05). These criteria are usually chosen to test for the model with best estimate of number of groups and predictors associated with them [15–17]. BIC is based, in part, on the likelihood function to measure the efficiency of the model to predict different groups in the data. Each is given a probability score for one's membership in the group. For each group, the mean of the probability scores of the individuals in the group is calculated and used as an indicator for adequate internal reliability if the value is more than 0.7 [18]. Odds of correct classification measure how improved the membership probability of individuals belonging to the in-group is as compared with other groups.

Sequence analysis in this research was conducted by software “*TraMineR*” in *R* statistical language [19]. Multiple clinical events of particular interest are fed into the program which allows it to search and identify order of them for each patient. The administrative data were manipulated and shuffled so that the time and diagnosis of every emergency hospital admission during the follow-up period were aligned in successive columns in the data [20]. The list of common causes of emergency readmissions is mentioned in Appendix. Each row in the dataset demonstrated the causes of hospital admissions that occurred with each patient, in a chronological order. Sequence analysis was performed on the transformed dataset as it can search, identify, and visualise sequences of events with each patient [19]. Each diagnosis was coded with a unique alphabet. For each patient, the string of sequence of alphabets was created based on their chronological order. Common strings of events were identified within each group.

## 3. Results

### 3.1. Elective AAA Repair

**Figure 1:**
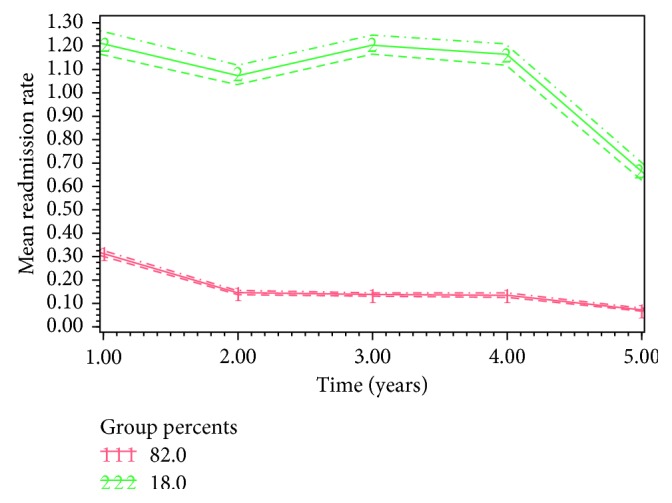
Trajectory pathways of subgroups of patients with elective AAA repair (the horizontal axis starts with annual readmission rate at year one, and the dotted lines represent 95% confidence intervals for each subgroup).

The best-fit model (BIC-61509, AIC-61474) classified the patient population (*n*=16,973) into 2 groups based on their nonelective readmissions: Group 1 (82.0%) and Group 2 (18%) (Figure 1). Group 1 had persistently low rate of readmission and, therefore, was classified as low-impact, while, group 2 had constant high rate of readmission and was labelled as high-impact.

**Figure 2:**
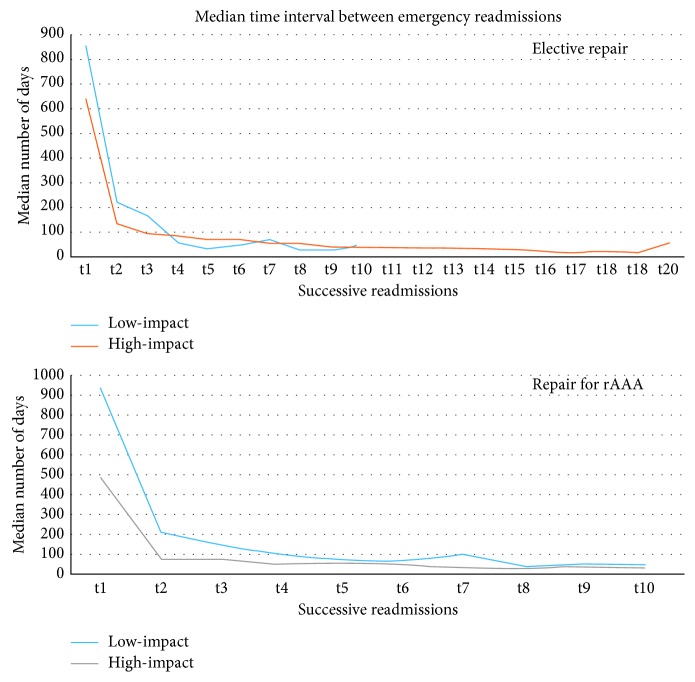
Median time interval between emergency readmissions from the time of AAA repair.

Within the patient population, the common causes of nonelective readmissions over 5-year period were respiratory tract infection (*n*=748, 7.7%), chest pain (*n*=543, 5.6%), aortic graft complications (*n*=465, 4.7%), gastrointestinal haemorrhage (*n*=462, 4.7%), and external injuries (*n*=461, 4.7%). Of the total population, 57.6% had emergency readmission (*n*=9791). Within low-impact users, 49.7% of them had emergency readmission (*n*=6918), none of these patients had multiple readmissions but had similar common causes of emergency readmissions. Of the high-impact users, 82.8% of them had emergency readmissions (*n*=2531). The common causes and sequences of readmissions are mentioned in Table 1. The time interval between each emergency readmission is displayed in Figure 2.

Subanalysis of elective open repair (*n*=10,801) showed that 10.2% of the patients were high-impact users. The most common causes of readmissions were respiratory tract infection (*n*=379 (9.1%)), aortic graft complications (*n*=214 (5.1%)), gastrointestinal haemorrhage (*n*=206 (4.9%)), unspecified chest pain (*n*=166 (3.9%)), and exacerbation of COPD (*n*=158 (3.7%)). The common sequences of readmissions are mentioned in Table 2. The most common causes of death were respiratory tract infection (16.1%), cancer (16.7%), heart failure (9.7%), renal failure (7.3%), and ischaemic heart disease (6.8%).

Subanalysis of elective EVAR (*n*=6172) showed that 13.5% of the patients were high-impact users. The most common causes of readmissions were respiratory tract infection (*n*=394 (9.2%)), exacerbation of COPD (*n*=248 (5.8%)), aortic graft complications (*n*=221 (5.1%)), heart failure (*n*=199 (4.6%)), and gastrointestinal haemorrhage (*n*=196 (4.5%)). Common sequences of readmissions are mentioned in Table 2. The most common causes of death were respiratory tract infection (17.2%), cancer (11.5%), heart failure (9.8%), renal failure (6.7%), and external injuries (6.2%).

### 3.2. Ruptured AAA Repair

**Figure 3:**
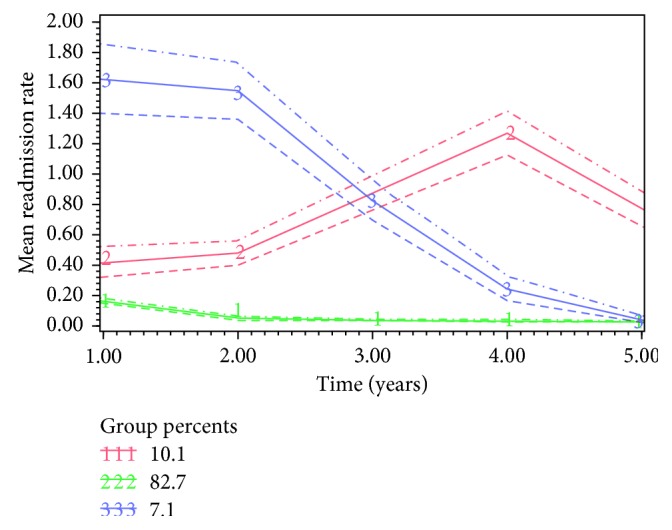
Trajectory pathways of subgroups of patients with ruptured AAA repair (the horizontal axis starts with annual readmission rate at year one, and the dotted lines represent 95% confidence intervals for each subgroup).

The best-fit model (BIC-9936, AIC-9895) classified the patient population (*n*=4144) into 3 subgroups based on their nonelective annual readmission rates: Group 1 (82.7%), Group 2 (10.1%), and Group 3 (7.2%) (Figure 3). Group 1 had persistently low rate of readmission and, therefore, was classified as low-impact. Those with high readmission rates (high-impact users) were part of Group 2 and Group 3. Group 2 included chronic high-impact users because they had persistently high readmission rate. Group 3 were short-term high-impact users who initially had high readmission rate but then had rapid decline in readmission rate.

Within patient population, the common causes of nonelective readmissions over 5-year period were respiratory tract infection (*n*=127, 8.4%), COPD (chronic obstructive pulmonary disease) (*n*=80, 5.3%), hypotension (*n*=77, 5.1%), gastrointestinal haemorrhage (*n*=70, 4.6%), and chest pain (*n*=70, 4.6%). Within low-impact users, the common causes of readmissions were respiratory tract infection (*n*=66, 7.9%), chest pain (*n*=45, 5.4%), hypotension (*n*=45, 5.4%), gastrointestinal haemorrhage (*n*=43, 5.2%), and external injury (*n*=41, 4.9%). No common sequences of multiple readmissions were identified among low-impact users. The common causes of readmissions and their distinct sequences among high-impact users are mentioned in Table 3.

**Figure 4:**
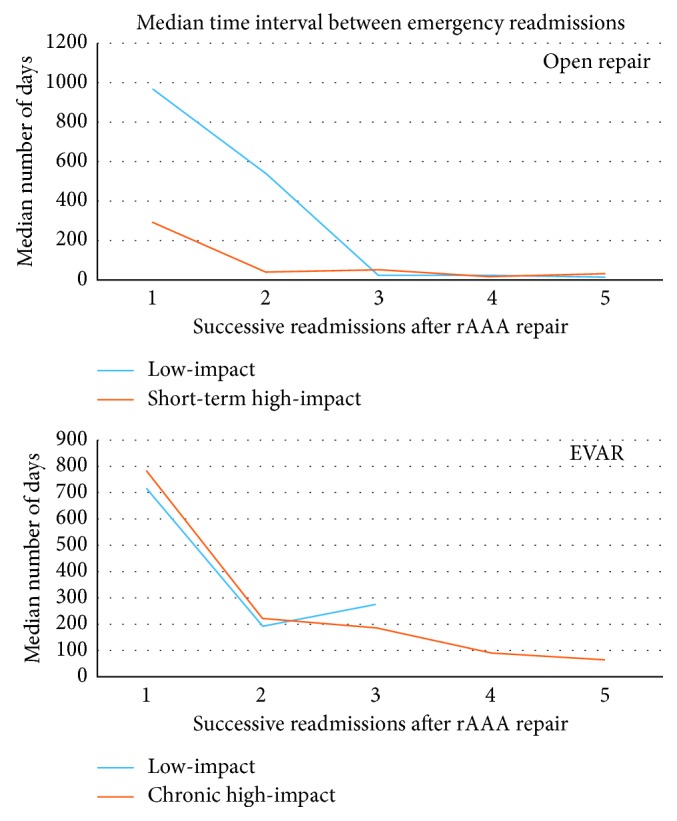
Time interval between first successive emergency readmissions following rAAA repair.

Subanalysis of open repair for rAAA (*n*=3877) showed that 6.6% of the patients were high-impact users. The most common causes of readmissions were exacerbation of COPD (*n*=36 (10.0%)), respiratory tract infection (*n*=30 (8.4%)), aortic graft complications (*n*=21 (5.8%)), and external injuries (*n*=16 (4.5%)). The common sequences of readmissions are mentioned in Table 4. The most common causes of death were respiratory tract infection (17.9%), cancer (11.9%), heart failure (8.9%), renal failure (16.4%), and sepsis (17.5%). The time interval between each successive readmission after rAAA repair is displayed in Figure 4.

Subanalysis of EVAR for rAAA (*n*=267) showed that 21.9% of the patients were high-impact users. The most common causes of readmissions were exacerbation of COPD (*n*=68 (17.5%)), aortic graft complications (*n*=44 (11.3%)), respiratory tract infection (*n*=32 (8.2%)), gastrointestinal haemorrhage (*n*=20 (5.1%)), and ischaemic heart disease (*n*=14 (3.6%)). The common sequences of readmissions are mentioned in Table 4. The most common causes of death were external injuries (31.2%), fractures (18.7%), aortic graft complications (12.5%), and respiratory tract infection (6.2%).

## 4. Discussion

Following AAA repair, high-impact users follow a distinct pathway of hospital care use. They have persistently high readmission rate as compared with low-impact users. They have significant number of patients with multiple emergency hospital admissions. Within rAAA repair patients, there was a third group, short-term high-impact, that initially had very high readmission rate followed by rapid decline. These patients were those with poor prognosis and did not survive after initial high readmission rate. High-impact users following AAA repair had repeated hospital admissions for cardiopulmonary conditions. The common causes of long-term readmissions in patient populations were respiratory tract infection, exacerbation of COPD, external injuries, and aortic graft complications.

There has been a debate about the proportion of emergency readmission that can be prevented in the community. The implementation of penalty for hospitals with higher than expected 30-day all-cause readmission rate among medical patients sparked research into preventative measures for causes of readmission. However, it was found that most of the readmissions were not preventable [21, 22]. It was not investigated what proportion of readmissions among vascular patients could be classified as preventable. This study has shown that patients with multiple hospital admissions mainly suffer from cardiopulmonary and aortic graft complications which can be potentially avoidable. Furthermore, 30-day readmission is routinely assessed in the clinical practice following this policy, and most research is conducted around it. It is based on earlier studies which showed that most of the readmissions occur within 30 days of discharge from the hospital. However, long-term follow-up of the patients refuted previous evidence and indicated that patients continue to have high readmission beyond 30 days and readmissions can occur even after one year. What happens to the readmission rate of high-impact users among vascular surgery patients? Is it different from the rest of the patients? Do they continue to have high readmission rate in the long-term? These questions are important, and the study attempted to answer them for better personalised care of the patients and possible role of management program among these patients.

All causes of emergency admissions during the 5-year period following rAAA repair were examined. Common causes of readmissions were cardiopulmonary conditions, aortic graft complications, and external injuries: these were common in all subgroups. Similar causes of readmission were found in earlier studies evaluating causes of short-term readmissions following AAA repair, but most of them are based on patients with elective repair [23]. The common causes of readmission after elective repair were wound complication, chest infection, sepsis, and myocardial infarction [24]. There was a higher rate of aortic graft complications and reintervention with EVAR use, but bowel obstruction, hernia repair, and gastrointestinal conditions were more common with open repair [24].

Since cardiopulmonary conditions are prevalent among sequences of multiple readmissions in high-impact users, a need for improved care to prevent exacerbation and progression of chronic cardiopulmonary conditions and infections in the community may be required to prevent multiple readmissions in high-impact users following AAA repair. The sequence analysis identified that multiple readmissions mainly consisted of a vicious cycle of COPD, respiratory tract infection, and ischaemic heart disease. It may suggest that the primary care team should be vigilant to assess patients once they are discharged back to the community after AAA repair. Meticulous preventative measures such as regular flu vaccination to prevent chest infection, secondary preventative medical therapy for ischaemic heart disease, and regular follow-up for COPD should be followed in these patients to prevent them from becoming high-impact. Moreover, the high-impact users had higher readmissions for aortic graft complications than the other group. In contrast, majority of low-impact users did not have any multiple readmissions.

All subgroups had similar common causes of readmissions, but high-impact users had significantly higher proportion of patients with multiple readmissions compared to other groups. The common sequences of causes of multiple readmissions in these patients consisted of exacerbation of COPD and chest infections. This was particularly important since it indicated that mere observation of common causes of readmission were similar in all subgroups. However, sequence analysis identified distinct sequences of readmissions that can be targeted by policymakers to prevent patients from having multiple readmissions.

Sequence analysis is a novel approach to study chronological order of events which can impact clinical outcome. This technique had been used in social science and psychology to understand pattern of events during the life-course of participants in the study. Previous studies have not evaluated temporal sequence of readmissions but only provided cross-sectional crude analysis of the common causes of readmissions. This technique can be applied to other adverse events or health-care services to assess deterioration in patient's health status. Hospital data did not contain information on the community events which led to hospital admissions. Further studies using primary care data will be helpful to understand all factors that lead to hospital readmissions among high-risk users.

This study had certain limitations despite the efforts to understand trends in readmission rates among subgroups of the patient population. The study was limited by the use of ICD and OPCS for identification of patient cohort, which is prone to coding errors in the administrative data collection [25]. With any retrospective cohort study, selection bias could not be ignored. The number of patients undergoing EVAR was small as compared with open repair. The patient cohort was selected to achieve follow-up of 5 years as it is counted as a minimum standard for the long-term follow-up by the Society for Vascular Surgeons [23]. During this period, the use of EVAR in rAAA was not widespread. Its use has increased in the last few years. The EVAR technique has significantly evolved with new catheters, graft stents, wires, and balloons for implantation. The clinicians are also more experienced in patient selection and procedural techniques [26]. Hence, analysis in the future is required to assess long-term morbidity associated with the procedure with advanced instrumentation. Furthermore, that the decision to perform EVAR is based on a complex interplay of availability of service, anatomical complexity, and patient comorbidity, and so there are biases in the selection of patients [27]. Therefore the differences between EVAR and open rAAA repair may be accounted for by this bias.

In conclusion, high-impact users form a significant number of patients who follow a distinct pathway of hospital care use following AAA repair. They mainly suffer from certain cardiopulmonary conditions in the community that lead to their recurrent hospital admissions. Prevention of these conditions can improve their health status. The potential role of cardiac rehabilitation after aneurysm repair and separate management pathway from the rest of the patient population should be further explored.

## Figures and Tables

**Table 1:** Common causes of emergency readmissions and their sequence among subgroups of patient populations. Aortic graft complications included graft infection, thrombosis, leak, and displacement.

High-impact group		
Common causes	Common sequences of readmissions	*N* (%)
RTI (*n*=207, 7.3%)	COPD → RTI	135 (4.7)
Aortic graft complications (*n*=171, 6.0%)	RTI → COPD	133 (4.7)
Chest pain (*n*=165, 5.8%)	Ischaemic heart disease → chest pain	93 (3.3)
Gastrointestinal haemorrhage (*n*=137, 4.8%)	Chest pain → ischaemic heart disease	83 (2.9)
COPD (*n*=136, 4.8%)	Urinary tract infection → RTI	74 (2.6)
External injuries (*n*=126, 4.7%)	RTI → COPD → COPD → RTI	67 (2.4)
Ischaemic heart disease (*n*=123, 4.3%)	COPD → RTI → RTI → COPD	64 (2.2)
Urinary tract infection (*n*=111, 3.9%)	External injury → RTI	62 (2.1)
Hypotension (*n*=102, 3.6%)	Chest pain → RTI	60 (2.1)
Heart failure (*n*=96, 3.4%)	RTI → heart failure	58 (2.0)

RTI: respiratory tract infection; COPD: Chronic obstructive pulmonary disease; IHD: ischaemic heart disease.

**Table 2:** Common sequences of causes of readmissions among high-impact users following elective open repair and EVAR.

High-impact group			
Elective open repair	*n* (%)	Elective EVAR	*n* (%)
COPD > RTI	18 (13.8)	Chest pain > IHD	11 (13.0)
RTI > COPD	15 (11.5)	IHD > chest pain	9 (10.7)
COPD > RTI > RTI > COPD	12 (9.2)	COPD > RTI	8 (9.5)
Chest pain > IHD	11 (8.4)	Chest pain > IHD > IHD > chest pain	7 (8.3)
IHD > chest pain	11 (8.4)	Chest pain > IHD	11 (13.0)

**Table 3:** Common causes and their sequences of readmissions among Group 1 and Group 3.

Group 2 (chronic high-impact)			Group 3 (short-term high-impact)		
Common causes (*N* (%))	Common sequences	*N* (%)	Common causes (*N* (%))	Common sequences	*N* (%)
RTI (34 (8.6))	COPD > RTI	22 (5.6)	RTI (27 (9.2))	COPD > RTI	17 (5.8)
COPD (26 (6.6))	RTI > COPD	21 (5.3)	COPD (22 (7.5))	UTI > RTI	14 (4.7)
External injuries (21 (5.3))	COPD > RTI > RTI > COPD	16 (4.1)	Aortic graft complications (18 (6.1))	COPD > COPD > RTI	10 (3.4)
Fractures (18 (4.5))	RTI > COPD > COPD > RTI	11 (2.8)	Hypotension (14 (4.7))	RTI > COPD	10 (3.4)
Aortic graft complications (18 (4.6))	COPD > COPD > RTI	10 (2.5)	Gastrointestinal haemorrhage (13 (4.4))	RTI > UTI	9 (3.1)
Hypotension (18 (4.5))	RTI > COPD > RTI > COPD	10 (2.5)	IHD (11 (3.7))	Dementia > RTI	8 (2.7)
UTI (18 (4.5))	RTI > UTI	10 (2.5)	rAAA (9 (3.1))	RTI > COPD > COPD > RTI	6 (2.0)

RTI, respiratory tract infection; COPD, chronic obstructive pulmonary disease; UTI, urinary tract infection; rAAA, ruptured abdominal aortic aneurysm.

**Table 4:** Common sequences of causes of readmissions among high-impact users following open repair and EVAR for rAAA.

High-impact group			
Open repair	*n* (%)	EVAR	*n* (%)
Chest infection > COPD	9 (18.6)	COPD > chest infection	3 (5.8)
COPD > chest infection > COPD	4 (8.6)	rAAA > graft complications	3 (5.8)
COPD > chest infection > chest infection > COPD	4 (8.6)	Unspecified chest pain > graft complications	2 (3.9)
Chest infection > COPD > COPD > chest infection	4 (8.6)	COPD > COPD > chest infection	2 (3.9)
Urine infection > chest infection	4 (8.6)	COPD > constipation	2 (3.9)

## Appendix

### All Causes of Emergency Readmissions following AAA Repair with ICD-10 Codes

Gastroenteritis: ‘A012', ‘A020', ‘A031', ‘A042', ‘A045', ‘A047', ‘A048', ‘A049', ‘A059', ‘A072', ‘A081', ‘A083', ‘A084', ‘A090', ‘A099', ‘A09X'

Sepsis: ‘A400', ‘A401', ‘A403', ‘A408', ‘A409', ‘A410', ‘A411', ‘A412', ‘A414', ‘A415', ‘A418', ‘A419'

Gastrointestinal cancers: ‘C153', ‘C154', ‘C155', ‘C159', ‘C160', ‘C161', ‘C162', ‘C163', ‘C165', ‘C166', ‘C169', ‘C170', ‘C172', ‘C179', ‘C180', ‘C181', ‘C182', ‘C183', ‘C184', ‘C185', ‘C186', ‘C187', ‘C189', ‘C19X', ‘C20X', ‘C211', ‘C220', ‘C221', ‘C224', ‘C229', ‘C23X', ‘C240', ‘C241', ‘C250', ‘C252', ‘C253', ‘C259'

Cardiothoracic cancers: ‘C340', ‘C341', ‘C342', ‘C343', ‘C349'

Prostate cancer: ‘C61X'

Renal cancer: ‘C670', ‘C671', ‘C672', ‘C674', ‘C678', ‘C679'

Secondary Carcinoma: ‘C780', ‘C782', ‘C784', ‘C785', ‘C786', ‘C787', ‘C788', ‘C791', ‘C793', ‘C794', ‘C795', ‘C798', ‘C799', ‘C800', ‘C809', ‘C80X'

Hodgkin's lymphoma: ‘C833'

Anaemia: ‘D500', ‘D501', ‘D508', ‘D509', ‘D648', ‘D649'

Neutropenia: ‘D70X'

Diabetes: ‘E100', ‘E101', ‘E105', ‘E109', ‘E110', ‘E111', ‘E112', ‘E113', ‘E114', ‘E115', ‘E116', ‘E119', ‘E160', ‘E161', ‘E162'

Disorder of mineral metabolism: ‘E831', ‘E834', ‘E835', ‘E86X', ‘E870', ‘E871', ‘E872', ‘E873', ‘E875', ‘E876', ‘E877', ‘E878'

Dehydration: ‘E86X'

Other disorders of fluids, electrolytes, acid-base balance: ‘E870', ‘E871', ‘E872', ‘E873', ‘E875', ‘E876', ‘E877', ‘E878'

Vascular dementia: ‘F011', ‘F012', ‘F013', ‘F018', ‘F019'

Unspecified dementia: ‘F03X'

Delirium: ‘F050', ‘F051', ‘F058', ‘F059'

Alcohol related mental health disorders: ‘F100', ‘F101', ‘F102', ‘F103', ‘F104', ‘F105', ‘F107', ‘F109'

Depression: ‘F320', ‘F321', ‘F322', ‘F323', ‘F329'

Anxiety: ‘F410', ‘F412', ‘F419'

Parkinson: ‘G20X'

Alzheimer's disease: ‘G300', ‘G301', ‘G308', ‘G309'

Epilepsy: ‘G400', ‘G401', ‘G402', ‘G403', ‘G406', ‘G407', ‘G408', ‘G409'

Migraine: ‘G431', ‘G438', ‘G439'

Transient ischaemic attack: ‘G450', ‘G451', ‘G453', ‘G454', ‘G458', ‘G459'

Hemi-plegia: ‘G819'

Hypertension: ‘I10X'

Hypertension due to chronic renal impairment: ‘I120', ‘I129'

Ischaemic heart disease: ‘I200', ‘I201', ‘I208', ‘I209', ‘I210', ‘I211', ‘I212', ‘I213', ‘I214', ‘I219', ‘I220', ‘I221', ‘I228', ‘I229', ‘I240', ‘I248', ‘I249', ‘I251', ‘I252', ‘I255', ‘I256', ‘I258', ‘I259'

Pulmonary embolism: ‘I260', ‘I269'

Endocarditis: ‘I330', ‘I339', ‘I38X'

Pericarditis: ‘I313', ‘I319'

Cardiac valvular disease: ‘I340', ‘I350', ‘I351', ‘I352', ‘I358'

Cardiac conduction disorders: ‘I440', ‘I441', ‘I442', ‘I447', ‘I451', ‘I452', ‘I453', ‘I455', ‘I458', ‘I459'

Cardiac arrest: ‘I460', ‘I469'

Cardiac arrhythmia disorders: ‘I470', ‘I471', ‘I472', ‘I479', ‘I490', ‘I493', ‘I494', ‘I495', ‘I498', ‘I499'

Atrial fibrillation: ‘I48X'

Heart failure: ‘I500', ‘I501', ‘I509'

Subarachnoid haemorrhage: ‘I601', ‘I602', ‘I603', ‘I604', ‘I605', ‘I608', ‘I609'

Intracerebral haemorrhage: ‘I610', ‘I611', ‘I612', ‘I613', ‘I614', ‘I615', ‘I616', ‘I618', ‘I619'

Subdural and extradural haemorrhage: ‘I620', ‘I621', ‘I629'

Ischaemic stroke: ‘I630', ‘I631', ‘I632', ‘I633', ‘I634', ‘I635', ‘I638', ‘I639'

Ill-defined stroke: ‘I64X'

Peripheral vascular disease: ‘I700', ‘I7000', ‘I701', ‘I702', ‘I7020', ‘I7021', ‘I708', ‘I709'

Abdominal aortic aneurysm dissection: ‘I710'

Ruptured thoracic aortic aneurysm: ‘I711'

Thoracic aortic aneurysm: ‘I712'

Ruptured abdominal aortic aneurysm: ‘I713', ‘I718'

Ruptured thoracoabdominal aortic aneurysm: ‘I715'

Aneurysms of iliac vessels: ‘I723'

Aneurysms of lower limb vessels: ‘I724'

Intermittent claudication: ‘I739'

Acute peripheral vascular condition: ‘I743', ‘I744', ‘I745', ‘I748'

Phlebitis: ‘I800', ‘I801', ‘I802', ‘I803', ‘I808', ‘I809'

Hypotension: ‘I951', ‘I952', ‘I958', ‘I959'

Respiratory tract infection: ‘J069', ‘J100', ‘J101', ‘J110', ‘J122', ‘J123', ‘J128', ‘J129', ‘J13X', ‘J14X', ‘J150', ‘J151', ‘J152', ‘J154', ‘J155', ‘J156', ‘J157', ‘J158', ‘J159', ‘J168', ‘J172', ‘J180', ‘J181', ‘J182', ‘J188', ‘J189', ‘J22X'

Chronic obstructive pulmonary disease: ‘J40X', ‘J42X', ‘J432', ‘J439', ‘J440', ‘J441', ‘J488', ‘J449'

Asthma: ‘J450', ‘J451', ‘J459', ‘J46X', ‘J47X'

Aspiration pneumonia: ‘J690', ‘J698'

Pulmonary oedema: ‘J81X'

Interstitial lung disease: ‘J841', ‘J848', ‘J849'

Pyothorax: ‘J860', ‘J869'

Pulmonary effusion: ‘J90X'

Pneumothorax: ‘J930', ‘J931', ‘J938', ‘J939'

Respiratory failure: ‘J960', ‘J961', ‘J969'

Esophagitis: ‘K20X'

Gastrooesophageal reflux disease: ‘K210', ‘K219'

Achalasia: ‘K220'

Oesophageal ulcer: ‘K221'

Oesophageal obstruction: ‘K222'

Oesophageal perforation: ‘K223'

Malery-Weis tear: ‘K226'

Gastrointestinal ulcer: ‘K260', ‘K261', ‘K262', ‘K263', ‘K264', ‘K265', ‘K266', ‘K267', ‘K269', ‘K270', ‘K274', ‘K275', ‘K277', ‘K279', ‘K283', ‘K284', ‘K285', ‘K289'

Gastroduodenitis: ‘K290', ‘K291', ‘K292', ‘K293', ‘K294', ‘K295', ‘K296', ‘K297', ‘K298', ‘K299', ‘K30X'

Appendicitis: ‘K350', ‘K351', ‘K352', ‘K353', ‘K358', ‘K359', ‘K36X', ‘K37X'

Hernia: ‘K400', ‘K402', ‘K403', ‘K404', ‘K409', ‘K413', ‘K414', ‘K419', ‘K420', ‘K421', ‘K429', ‘K430', ‘K431', ‘K439', ‘K440', ‘K449', ‘K450', ‘K451', ‘K458', ‘K449', ‘K450', ‘K451', ‘K458', ‘K460', ‘K469'

Noninfective colitis: ‘K500', ‘K501', ‘K508', ‘K509', ‘K510', ‘K512', ‘K513', ‘K514', ‘K515', ‘K518', ‘K519', ‘K520', ‘K521', ‘K522', ‘K523', ‘K528', ‘K529'

Acute mesenteric artery ischaemia: ‘K550', ‘K551', ‘K552', ‘K558', ‘K559', ‘K580', ‘K589'

Intestinal obstruction: ‘K560', ‘K561', ‘K562', ‘K563', ‘K564', ‘K565', ‘K566', ‘K567'

Diverticular disease: ‘K570', ‘K571', ‘K572', ‘K573', ‘K574', ‘K575', ‘K578', ‘K579'

Constipation: ‘K590'

Perianal abscess: ‘K610', ‘K611', ‘K612', ‘K613'

Rectal bleeding: ‘K620', ‘K621', ‘K622', ‘K623', ‘K624', ‘K625', ‘K626', ‘K627', ‘K628', ‘K629'

Gastrointestinal perforation: ‘K630', ‘K631', ‘K632', ‘K633', ‘K634', ‘K635', ‘K638', ‘K639'

Peritonitis: ‘K650', ‘K658', ‘K659', ‘K660', ‘K661', ‘K668', ‘K669'

Alcohol liver disease: ‘K700', ‘K701', ‘K703', ‘K704', ‘K709'

Disorders of gall bladder: ‘K800', ‘K801', ‘K802', ‘K803', ‘K804', ‘K805', ‘K808', ‘K810', ‘K811', ‘K818', ‘K819'

Disorders of bile duct: ‘K830', ‘K831', ‘K832', ‘K833', ‘K835', ‘K838', ‘K839'

Disorders of pancreas: ‘K850', ‘K851', ‘K852', ‘K853', ‘K858', ‘K859', ‘K85X'

Postoperative gastrointestinal complications (obstruction, malabsorption, pain, vomiting): ‘K900', ‘K904', ‘K908', ‘K909', ‘K910', ‘K912', ‘K913', ‘K914', ‘K918', ‘K919'

Gastrointestinal bleeding: ‘K920', ‘K921', ‘K922', ‘K928', ‘K929'

Skin infection: ‘L020', ‘L021', ‘L022', ‘L023', ‘L024', ‘L029', ‘L030', ‘L031', ‘L032', ‘L033', ‘L038', ‘L039', ‘L088', ‘L089'

Lower limb ulcers: ‘L97X'

Gout: ‘M100', ‘M1000', ‘M1004', ‘M1006', ‘M1007', ‘M1020', ‘M1034', ‘M1037', ‘M109', ‘M1090', ‘M1092', ‘M1093', ‘M1094', ‘M1095', ‘M1096', ‘M1097', ‘M1099'

Disorders of peripheral joints: ‘M200', ‘M201', ‘M203', ‘M204', ‘M205', ‘M206', ‘M210', ‘M2106', ‘M2107', ‘M2115', ‘M2116', ‘M2117', ‘M212', ‘M2122', ‘M2123', ‘M2124', ‘M2127', ‘M2129', ‘M213', ‘M2133', ‘M2137', ‘M214', ‘M215', ‘M2154', ‘M2157', ‘M216', ‘M2167', ‘M2176', ‘M2184', ‘M2190', ‘M2194', ‘M2196', ‘M2197', ‘M220', ‘M222', ‘M224', ‘M2309', ‘M2316', ‘M232', ‘M2320', ‘M2321', ‘M2322', ‘M2323', ‘M2324', ‘M2325', ‘M2326', ‘M2327', ‘M2329', ‘M233', ‘M2330', ‘M2331', ‘M2332', ‘M2333', ‘M2335', ‘M2336', ‘M2339', ‘M234', ‘M235', ‘M2351', ‘M238', ‘M2381', ‘M2382', ‘M2382', ‘M2386', ‘M2389', ‘M239', ‘M2396', ‘M2411', ‘M2412', ‘M2413', ‘M2415', ‘M2416', ‘M2417', ‘M2421', ‘M2426', ‘M2431', ‘M244', ‘M2441', ‘M2442', ‘M2445', ‘M2448', ‘M2450', ‘M2451', ‘M2452', ‘M2453', ‘M2454', ‘M2455', ‘M2456', ‘M2457', ‘M2466', ‘M2468', ‘M248', ‘M2483', ‘M2485', ‘M2486', ‘M250', ‘M2500', ‘M2501', ‘M2502', ‘M2503', ‘M2504', ‘M2505', ‘M2506', ‘M2507', ‘M2515', ‘M2521', ‘M2526', ‘M253', ‘M2531', ‘M2532', ‘M2533', ‘M2536', ‘M2537', ‘M254', ‘M2540', ‘M2541', ‘M2542', ‘M2543', ‘M2544', ‘M2545', ‘M2546', ‘M2547', ‘M255', ‘M2550', ‘M2551', ‘M2552', ‘M2553', ‘M2554', ‘M2555', ‘M2556', ‘M2557', ‘M2558', ‘M2559', ‘M256', ‘M2560', ‘M2561', ‘M2562', ‘M2563', ‘M2564', ‘M2565', ‘M2566', ‘M2567', ‘M257', ‘M2575', ‘M2576', ‘M2577', ‘M2578', ‘M2581', ‘M2583', ‘M2584', ‘M2585', ‘M2586', ‘M2587', ‘M2591', ‘M2591', ‘M2595', ‘M2596', ‘M2597', ‘M2598', ‘M301', ‘M311', ‘M313', ‘M314', ‘M315', ‘M316', ‘M317', ‘M318', ‘M319', ‘M320', ‘M321', ‘M329', ‘M331', ‘M332', ‘M341', ‘M348', ‘M349', ‘M350', ‘M352', ‘M353', ‘M354', ‘M357', ‘M358', ‘M359'

‘M4015', ‘M402', ‘M4023', ‘M4025', ‘M4026', ‘M4029', ‘M4056', ‘M4029', ‘M4056', ‘M4186', ‘M419', ‘M4190', ‘M4192', ‘M4194', ‘M4195', ‘M4196', ‘M4197', ‘M4199', ‘M4297', ‘M4300', ‘M4302', ‘M4306', ‘M4307', ‘M4309', ‘M431”M4312', ‘M4316', ‘M4317', ‘M4327', ‘M436', ‘M45X', ‘M45X0', ‘M45X2', ‘M45X9', ‘M461', ‘M4626', ‘M4628', ‘M4632', ‘M4636', ‘M464', ‘M4640', ‘M4642', ‘M4644', ‘M4645', ‘M4646', ‘M4650', ‘M4656', ‘M469', ‘M4690', ‘M4692', ‘M4696', ‘M4697', ‘M470', ‘M471', ‘M4711', ‘M4712', ‘M4716', ‘M472', ‘M4720', ‘M4722', ‘M4726', ‘M4727', ‘M478', ‘M4780', ‘M4781', ‘M4782', ‘M4783', ‘M4784', ‘M4785', ‘M4786', ‘M4787', ‘M4788', ‘M4789', ‘M479', ‘M4790', ‘M4792', ‘M4794', ‘M4796', ‘M4797', ‘M4798', ‘M4799', ‘M480', ‘M4800', ‘M4802', ‘M4805', ‘M4806', ‘M4807', ‘M4808', ‘M4809', ‘M4819', ‘M4844', ‘M4846', ‘M4848', ‘M485', ‘M4850', ‘M4852', ‘M4854', ‘M4855', ‘M4856', ‘M4858', ‘M4859', ‘M4884', ‘M4887', ‘M489', ‘M4895', ‘M4896', ‘M4950', ‘M4956', ‘M500', ‘M501', ‘M502', ‘M503', ‘M510', ‘M511', ‘M512', ‘M513', ‘M518', ‘M519', ‘M532', ‘M5327', ‘M533', ‘M5338', ‘M5380', ‘M5386', ‘M540', ‘M541', ‘M5412', ‘M5414', ‘M5414', ‘M5415', ‘M5416', ‘M5417', ‘M5418', ‘M5419', ‘M542', ‘M5420', ‘M5421', ‘M5422', ‘M5423', ‘M5429', ‘M543', ‘M5430', ‘M5436', ‘M5437', ‘M5438', ‘M5439', ‘M544', ‘M5440', ‘M5446', ‘M5447', ‘M5449', ‘M545', ‘M5450', ‘M5455', ‘M5456', ‘M5457', ‘M5458', ‘M5459', ‘M546', ‘M5464', ‘M548', ‘M5480', ‘M5484', ‘M5485', ‘M5487', ‘M5488', ‘M5489', ‘M549', ‘M5490', ‘M5492', ‘M5494', ‘M5495', ‘M5496', ‘M5497', ‘M5498', ‘M5499'

Pathological fracture: ‘M8008', ‘M8045', ‘M8048', ‘M8058', ‘M808', ‘M8080', ‘M8088', ‘M809', ‘M8090', ‘M8095', ‘M8096', ‘M8097', ‘M8098', ‘M8099'

Haematuria: ‘N020', ‘N022', ‘N028', ‘N029'

Obstructive nephropathy: ‘N131', ‘N132', ‘N133', ‘N134', ‘N135', ‘N136', ‘N137', ‘N138', ‘N139'

Acute renal failure: ‘N170', ‘N171', ‘N178', ‘N179', ‘N19X'

Chronic renal failure: ‘N180', ‘N182', ‘N183', ‘N184', ‘N185', ‘N188', ‘N189'

Urolithiasis: ‘N200', ‘N201', ‘N202', ‘N209', ‘N210', ‘N211', ‘N218', ‘N219', ‘N23X'

Urinary tract infection: ‘N10', ‘N12', ‘N151', ‘N159', ‘N158', ‘N160', ‘N288', ‘N300', ‘N302', ‘N304', ‘N303', ‘N308', ‘N309', ‘N340', ‘N341', ‘N342', ‘N370', ‘N390', ‘N410', ‘N450', ‘N459', ‘N47', ‘N511', ‘A540', ‘A560', ‘A562', ‘B374'

Benign prostate hypertrophy: ‘N40X'

Epidydimoorchitis: ‘N450', ‘N459'

Unspecified bradycardia: ‘R000', ‘R001', ‘R002', ‘R008'

Unspecified haemoptysis: ‘R040', ‘R042'

Unspecified shortness of breath: ‘R060', ‘R061', ‘R062', ‘R063', ‘R064', ‘R065', ‘R066', ‘R068'

Unspecified chest pain: ‘R070', ‘R071', ‘R072', ‘R073', ‘R074'

Unspecified nausea and vomiting: ‘R11X'

Unspecified dysphagia: ‘R13X'

Unspecified skin lump: ‘R220', ‘R221', ‘R222', ‘R223', ‘R224', ‘R227', ‘R229'

Unspecified abnormal gait: ‘R260', ‘R261', ‘R262', ‘R263', ‘R268'

Unspecified musculoskeletal pain: ‘R290', ‘R293', ‘R296', ‘R298'

Unspecified haematuria: ‘R31X'

Urinary retention: ‘R33X'

Amnesia: ‘R410'

Unspecified dizziness: ‘R42X'

Speech disorders: ‘R470', ‘R471', ‘R478'

Unspecified headache: ‘R51X'

Unspecified malaise: ‘R53X'

Unspecified senility: ‘R54X'

Unspecified convulsions: ‘R560', ‘R568'

Syncope: ‘R55X'

Hip fracture: ‘S720', ‘S7200', ‘S721', ‘S7210'

Lower limb fracture: ‘S820', ‘S8200', ‘S821', ‘S8210'

Upper limb fracture: ‘S520', ‘S5200', ‘S521', ‘S522', ‘S5241', ‘S525', ‘S5250', ‘S5251', ‘S526', ‘S5260', ‘S5261', ‘S5270', ‘S5280'

Spinal fracture: ‘S320', ‘S3200', ‘S3210', ‘S323', ‘S324', ‘S3240', ‘S3241', ‘S325', ‘S3250', ‘S3270', ‘S3280'

External injury: ‘S000', ‘S001', ‘S002', ‘S003', ‘S004', ‘S005', ‘S006', ‘S007', ‘S008', ‘S009', ‘S010', ‘S011', ‘S012', ‘S013', ‘S014', ‘S015', ‘S016', ‘S017', ‘S018', ‘S019', ‘S020', ‘S0200', ‘S021', ‘S0210', ‘S022', ‘S0220', ‘S0221', ‘S0230', ‘S0231', ‘S0240', ‘S0250', ‘S0260', ‘S0270', ‘S0280', ‘S0290', ‘S0600', ‘S0620', ‘S0630', ‘S0641', ‘S0640', ‘S0650', ‘S0680', ‘S0660', ‘S0650', ‘S0651', ‘S0680', ‘S0681', ‘S0690', ‘S0691', ‘S023', ‘S024', ‘S025', ‘S026', ‘S027', ‘S028', ‘S029', ‘S030', ‘S031', ‘S032', ‘S033', ‘S034', ‘S035', ‘S040', ‘S041', ‘S042', ‘S043', ‘S044', ‘S045', ‘S046', ‘S047', ‘S048', ‘S049', ‘S050', ‘S051', ‘S052', ‘S053', ‘S054', ‘S055', ‘S056', ‘S057', ‘S058', ‘S059', ‘S060', ‘S061', ‘S062', ‘S063', ‘S064', ‘S065', ‘S066', ‘S067', ‘S068', ‘S069', ‘S070', ‘S071', ‘S078', ‘S079', ‘S080', ‘S081', ‘S088', ‘S089', ‘S090', ‘S091', ‘S092', ‘S079', ‘S080', ‘S081', ‘S088', ‘S089', ‘S090', ‘S091', ‘S092', ‘S097', ‘S098', ‘S099', ‘S100', ‘S101', ‘S107', ‘S108', ‘S109', ‘S110', ‘S111', ‘S112', ‘S118', ‘S119', ‘S120', ‘S121', ‘S122', ‘S127', ‘S128', ‘S129', ‘S130', ‘S131', ‘S132', ‘S133', ‘S134', ‘S135', ‘S136', ‘S140', ‘S141', ‘S142', ‘S143', ‘S144', ‘S145', ‘S146', ‘S150', ‘S150', ‘S151', ‘S152', ‘S153', ‘S157', ‘S158', ‘S159', ‘S16', ‘S170', ‘S178', ‘S179', ‘S18', ‘S197', ‘S198', ‘S199', ‘S1200', ‘S1210', ‘S1220', ‘S1270', ‘S1290', ‘S2220', ‘S2230', ‘S2240', ‘S2250', ‘S2680', ‘S2690', ‘S2700', ‘S2710', ‘S2720', ‘S2210', ‘S2730', ‘S200', ‘S201', ‘S202', ‘S203', ‘S204', ‘S205', ‘S206', ‘S207', ‘S208', ‘S21', ‘S210', ‘S211', ‘S212', ‘S217', ‘S218', ‘S219', ‘S220', ‘S221', ‘S222', ‘S223', ‘S224', ‘S225', ‘S228', ‘S229', ‘S230', ‘S231', ‘S232', ‘S233', ‘S234', ‘S235', ‘S240', ‘S241', ‘S242', ‘S243', ‘S244', ‘S245', ‘S246', ‘S250', ‘S251', ‘S252', ‘S253', ‘S254', ‘S255', ‘S257', ‘S258', ‘S259', ‘S260', ‘S268', ‘S269', ‘S270', ‘S271', ‘S272', ‘S273', ‘S274', ‘S275', ‘S276', ‘S277', ‘S278', ‘S279', ‘S280', ‘S281', ‘S290', ‘S297', ‘S298', ‘S299', ‘S300', ‘S301', ‘S302', ‘S3200', ‘S3220', ‘S3230', ‘S3240', ‘S3280', ‘S3250', ‘S3600', ‘S3601', ‘S3601', ‘S3650', ‘S3660', ‘S3671', ‘S3680', ‘S3700', ‘S3720', ‘S3730', ‘S3790', ‘S307', ‘S308', ‘S309', ‘S310', ‘S311', ‘S312', ‘S313', ‘S314', ‘S315', ‘S316', ‘S317', ‘S318', ‘S320', ‘S321', ‘S322', ‘S323', ‘S324', ‘S325', ‘S326', ‘S327', ‘S328', ‘S330', ‘S331', ‘S332', ‘S333', ‘S334', ‘S335', ‘S336', ‘S337', ‘S340', ‘S341', ‘S342', ‘S343', ‘S344', ‘S345', ‘S346', ‘S347', ‘S348', ‘S350', ‘S351', ‘S352', ‘S353', ‘S354', ‘S355', ‘S356', ‘S357', ‘S358', ‘S359', ‘S360', ‘S361', ‘S362', ‘S363', ‘S364', ‘S365', ‘S367', ‘S368', ‘S369', ‘S370', ‘S371', ‘S372', ‘S373', ‘S374', ‘S375', ‘S376', ‘S377', ‘S378', ‘S380', ‘S381', ‘S382', ‘S383', ‘S390', ‘S396', ‘S397', ‘S398', ‘S399', ‘S500', ‘S501', ‘S507', ‘S508', ‘S509', ‘S510', ‘S517', ‘S518', ‘S519', ‘S520', ‘S521', ‘S522', ‘S523', ‘S524', ‘S525', ‘S526', ‘S5200', ‘S5201', ‘S5210', ‘S5220', ‘S5221', ‘S5230', ‘S5240', ‘S5250', ‘S5251', ‘S5260', ‘S5261', ‘S5270', ‘S5280', ‘S5290', ‘S527', ‘S528', ‘S529', ‘S530', ‘S531', ‘S532', ‘S533', ‘S534', ‘S540', ‘S541', ‘S542', ‘S543', ‘S547', ‘S548', ‘S549', ‘S550', ‘S551', ‘S557', ‘S558', ‘S559', ‘S560', ‘S561', ‘S562', ‘S563', ‘S564', ‘S565', ‘S566', ‘S567', ‘S568', ‘S559', ‘S560', ‘S561', ‘S562', ‘S563', ‘S564', ‘S565', ‘S566', ‘S567', ‘S568', ‘S570', ‘S578', ‘S579', ‘S580', ‘S581', ‘S589', ‘S597', ‘S598', ‘S599', ‘S600', ‘S601', ‘S602', ‘S6200', ‘S6210', ‘S6220', ‘S6230', ‘S6231', ‘S6240', ‘S6250', ‘S6251', ‘S6260', ‘S6261', ‘S6270', ‘S6280', ‘S607', ‘S608', ‘S609', ‘S610', ‘S611', ‘S617', ‘S618', ‘S619', ‘S620', ‘S621', ‘S622', ‘S623', ‘S624', ‘S625', ‘S626', ‘S627', ‘S628', ‘S630', ‘S631', ‘S632', ‘S633', ‘S634', ‘S635', ‘S636', ‘S637', ‘S640', ‘S641', ‘S642', ‘S643', ‘S644', ‘S647', ‘S648', ‘S649', ‘S650', ‘S651', ‘S652', ‘S653', ‘S654', ‘S655', ‘S656', ‘S657', ‘S658', ‘S659', ‘S660', ‘S661', ‘S662', ‘S663', ‘S664', ‘S665', ‘S666', ‘S667', ‘S668', ‘S669', ‘S670', ‘S678', ‘S680', ‘S681', ‘S682', ‘S683', ‘S684', ‘S688', ‘S689', ‘S697', ‘S698', ‘S699', ‘S700', ‘S701', ‘S707', ‘S708', ‘S710', ‘S718', ‘S720', ‘S721', ‘S722', ‘S723', ‘S724', ‘S727', ‘S728', ‘S729', ‘S730', ‘S731', ‘S740', ‘S741', ‘S742', ‘S747', ‘S749', ‘S750', ‘S751', ‘S752', ‘S757', ‘S758', ‘S759', ‘S760', ‘S761', ‘S762', ‘S763', ‘S764', ‘S767', ‘S770', ‘S771', ‘S772', ‘S780', ‘S781', ‘S789', ‘S797', ‘S798', ‘S799', ‘S7200', ‘S7201', ‘S7210', ‘S7211', ‘S7220', ‘S7230', ‘S7240', ‘S7241', ‘S7270', ‘S7280', ‘S7290', ‘S800', ‘S801', ‘S807', ‘S808', ‘S809', ‘S810', ‘S817', ‘S818', ‘S819', ‘S820', ‘S821', ‘S822', ‘S8200', ‘S8201', ‘S8210', ‘S8211', ‘S8220', ‘S8221', ‘S8230', ‘S8231', ‘S8240', ‘S8250', ‘S8251', ‘S8260', ‘S8261', ‘S8270', ‘S8271', ‘S8280', ‘S8281', ‘S823', ‘S824', ‘S825', ‘S826', ‘S827', ‘S828', ‘S829', ‘S830', ‘S831', ‘S832', ‘S833', ‘S834', ‘S835', ‘S836', ‘S837', ‘S840', ‘S841', ‘S842', ‘S847', ‘S848', ‘S849', ‘S850', ‘S851', ‘S852', ‘S853', ‘S854', ‘S855', ‘S856', ‘S857', ‘S858', ‘S859', ‘S860', ‘S861', ‘S862', ‘S863', ‘S867', ‘S868', ‘S869', ‘S870', ‘S878', ‘S880', ‘S881', ‘S889', ‘S900', ‘S901', ‘S902', ‘S903', ‘S907', ‘S908', ‘S909', ‘S910', ‘S911', ‘S912', ‘S913', ‘S917', ‘S920', ‘S921', ‘S922', ‘S923', ‘S924', ‘S925', ‘S926', ‘S927', ‘S929', ‘S930', ‘S931', ‘S932', ‘S933', ‘S934', ‘S935', ‘S936', ‘S940', ‘S941', ‘S942', ‘S943', ‘S947', ‘S948', ‘S949', ‘S950', ‘S951', ‘S952', ‘S957', ‘S958', ‘S959', ‘S960', ‘S961', ‘S962', ‘S967', ‘S968', ‘S969', ‘S970', ‘S971', ‘S978', ‘S980', ‘S981', ‘S982', ‘S983', ‘S984', ‘S997', ‘S998', ‘S999', ‘S9200', ‘S9210', ‘S9220', ‘S9230', ‘S9240', ‘S9241', ‘S9250', ‘S9251', ‘S9270', ‘T181', ‘T212', ‘T390', ‘T391', ‘T402', ‘T406', ‘T404', ‘T424', ‘T426', ‘T455', ‘T460', ‘T464', ‘T598', ‘T68X', ‘T783', ‘T784', ‘T802', ‘Z038', ‘Z049', ‘Z090', ‘Z092', ‘Z098', ‘Z136', ‘Z139', ‘Z436', ‘Z452', ‘Z466', ‘Z458', ‘Z739'

Aortic graft complications (leak, thrombosis, infection, migration): ‘T810', ‘T811', ‘T812', ‘T813', ‘T814', ‘T815', ‘T816', ‘T817', ‘T818', ‘T819', ‘T820', ‘T821', ‘T822', ‘T823', ‘T824', ‘T825', ‘T826', ‘T827', ‘T828', ‘T829', ‘T830', ‘T831', ‘T832', ‘T833', ‘T834', ‘T835', ‘T836', ‘T838', ‘T839', ‘T840', ‘T841', ‘T842', ‘T843', ‘T844', ‘T845', ‘T846', ‘T847', ‘T848', ‘T849', ‘T850', ‘T851', ‘T852', ‘T853', ‘T854', ‘T855', ‘T856', ‘T857', ‘T858', ‘T859', ‘T860', ‘T861', ‘T864', ‘T868', ‘T873', ‘T874', ‘T875', ‘T876', ‘T884', ‘T885', ‘T886', ‘T887', ‘T888', ‘T889'
